# Reconstruction of deep and perforating corneal defects in dogs—A review (Part III/III): The use of corneal sutures and reporting of ocular discomfort

**DOI:** 10.1111/vop.13285

**Published:** 2024-10-01

**Authors:** R. F. Sanchez

**Affiliations:** ^1^ Specialistische Dierenkliniek Utrecth (SDU‐AniCura) Utrecht The Netherlands

**Keywords:** buried suture, corneal reconstruction, deep‐superficial‐superficial‐deep, microsuture, suture track, suture tug

## Abstract

The surgical reconstruction of severe corneal disease is a common and crucial component of the clinical practice of veterinary ophthalmology. The first part of the present review described procedures that utilize autogenous ocular tissues, homologous donor tissues, and heterologous donor tissues in dogs, while the second part reviewed the use of biomaterials and keratoprosthetics in this species. This third part is dedicated to the review of the use of corneal sutures including suture type and suture pattern in corneal reconstruction of small animals including dogs and cats. The review also focused on the way studies report postoperative ocular discomfort/pain and how this is treated. Lastly, the author briefly presents the simple but effective techniques available to bury corneal knots for corneal reconstructive surgery in small animal patients, such as the “tugging” and “deep‐superficial‐superficial‐deep” methods for simple interrupted sutures, and the adaptation of the latter for simple continuous sutures.

## 1 INTRODUCTION

Corneal ulcerative disease (CUD) comprises a painful and potentially devastating group of conditions that often require a surgical approach.^1^ Surgical reconstruction of corneal wounds is recommended when the ulcer bed has reached 50% of stromal depth, while corneal lacerations without stromal loss might be directly closed with sutures and those with stromal loss caused by keratomalacia, trauma, or keratectomy might require reconstruction.^1^

Corneal reconstructive surgery for small animals aims to save the globe and, if possible, to preserve transmission through the visual axis.^1^ A variety of corneal reconstructive techniques have been described for this purpose and include the use of the patient's own ocular tissues,^2^, ^3^, ^4^, ^5^, ^6^, ^7^, ^8^, ^9^, ^10^, ^11^, ^12^, ^13^, ^14^, ^15^, ^16^, ^17^, ^18^, ^19^, ^20^, ^21^ donor ocular tissue from a variety of sources,^22^, ^23^, ^24^, ^25^, ^26^, ^27^, ^28^, ^29^ commercially available biomaterials,^19^, ^30^, ^31^, ^32^, ^33^, ^34^, ^35^, ^36^, ^37^, ^38^, ^39^, ^40^ biomaterials from other sources,^41^, ^42^ or a combination of two of these.^13^, ^28^, ^43^

Generally speaking, surgeons use corneal microsutures to appose the edges of a graft, flap, or biomaterial to those of the recipient cornea, to directly suture a laceration, or to close surgical wounds in cataract surgery.^44^ The microsuture recommended for corneal use in veterinary ophthalmology include 9–0 or 10–0, monofilament, absorbable or nonabsorbable suture, depending on the preference of the surgeon, while recommended suture patterns include simple interrupted and simple continuous.^44^, ^45^ Suture knots typically contain a double “apposing throw,” followed by a single “locking throw” and an additional single “locking throw.”^44^, ^45^ These knots, though small, are hard and irregular foreign bodies and, as such, they lead to palpebral and third eyelid conjunctival irritation during blinking and ocular movement.^45^, ^46^ Ocular pain in corneal disease may be directly caused by keratitis and reflex uveitis,^1^ which are conditions amenable to medical therapy and, in some cases, a combination of surgery and medical therapy. It would be logical to assume the irritation caused by external microsuture knots during blinking might exacerbate the discomfort caused by an already existing keratitis and/or reflex uveitis and possibly worsen these conditions by increasing inflammation locally. However, the potential source of pain/discomfort caused by the use of external microsuture knots risks being inadvertently ignored by veterinary corneal surgeons if it is not generally discussed in corneal reconstruction studies. Unfortunately, differentiating between potential irritation or pain caused by a knot left on the ocular surface or a pre‐existing keratitis and/or reflex uveitis carries numerous challenges. An important one is the lack of an ocular pain scoring system for use in small animal patients that veterinary ophthalmologists may rely on. Having said this, how often veterinary corneal surgeons report using knot burying techniques in the veterinary literature and whether or not this reflects how frequently these techniques might be mentioned in veterinary ophthalmology textbooks remains unknown.

It is important to have a general overview of the use of corneal sutures in corneal reconstruction of small animals, the way authors report postoperative ocular discomfort/pain, and how potential discomfort/pain is treated. However, there are no reviews in the veterinary literature with a focus on the use of suture material, suture pattern, and suture knots used in small animal corneal reconstructive surgery or the manner in which studies report and treat ocular discomfort/pain after corneal reconstructive surgery.

The aims of this article are to review the use of corneal sutures in corneal reconstruction of dogs in the veterinary literature, whether or not surgeons report postoperative patient comfort in these studies, and to comment on the techniques available to bury corneal knots for corneal reconstructive surgery. The goals include to summarize the reported use of corneal suturing techniques in veterinary ophthalmic practice, to identify areas where key information might be missing from studies that could inform the planning of patient record keeping and future studies, and to highlight the presence of knowledge gaps that deserve further attention.

## 2 MATERIALS AND METHODS

A literature review was performed to identify source material using the same search strategy defined in the first part of this review on corneal reconstructive techniques in dogs. The articles in the resulting list were reviewed to assess the number of patients, and species included, suture size, suture material, suture pattern, type of knot configuration used, and if the knot was buried or if it remained on the ocular surface (i.e., either because it was described in the text and/or shown in clinical photographs). This was expressed as a percentage out of the total number of articles (i.e., number of articles that reported the knot configuration used), or out of the total number of times an category was counted irrespective of the total number of articles (i.e., suture size, type and pattern used, and if knots were buried or not, as one article might have included more than one of these categories). When authors mentioned in the text that suture knots were left on the surface, or when at least some of the knots shown on the images provided by the authors were external, the knots were categorized as being external independent of the possible presence of buried knots combined with superficial knots.

The articles were also reviewed to construct a separate overview of a variety of aspects to do with the reporting of preoperative and postoperative discomfort/pain and the medication used to control it, such as an oral non‐steroidal anti‐inflammatory drug (NSAID), or non‐medical methods (e.g., tarsorrhaphy and bandage lens), and the use of topical drugs that might have been used to control iridociliary body spasm in the days that followed the surgery. This was expressed as a percentage out of the total number of articles.

A review of veterinary ophthalmic textbooks that contained surgical chapters was also carried out to determine if the use of buried knots was mentioned and/or recommended and, if so, what techniques were presented to carry this out. In addition to textbooks, single‐topic journals with an issue on small animal ophthalmic surgery as one of the dedicated issues were included. In cases where more than one edition existed, only the latest edition was included.

## 3 RESULTS

### 3.1 Number of peer‐reviewed articles and total number of canine cases included

The peer‐reviewed veterinary literature written in English in the last 61 years (i.e., start of 1962 to end of 2023) that focused on corneal reconstruction for spontaneous CUD of various causes in canine patients contained 30 papers (Table 1). These were reviewed as described in the materials and methods section. The study by A. M. Lavignette (Small Animal Clinician, 1962)^2^ was included in the list due to its historical significance, but the cases were excluded from the count, as the surgical techniques and the suture material were not later used in dogs. There were 1272 canine eyes in the remaining 29 articles (Table 1). It should be noted that some of the articles included feline eyes (i.e., 7 of the 14 articles on the use of the patient's own tissues, 3 out of the 4 articles on the use of donor tissue, and 6 out of the 11 articles on the use of biomaterials, see Table 1) and the authors reported the results together with those of the canine eyes, which means they could not be separated.

**TABLE 1:** Summary of the most important findings of the review of the use of corneal sutures and reporting of ocular discomfort.

No.	Reference and type of surgery	No. of eyes and species	Treatments included	Suture type and pattern used, and what was reported more often (i.e., absorbable or nonabsorbable)	Position of suture knots	Use of cycloplegic/mydriatic	Additional pain control postoperatively	No. of articles that mentioned pain/discomfort, and the section/s in which this was mentioned in the article.
***n* = 14**	**Patients' own tissues:** (Lavignette, 1962) Parsahll, 1973 Peiffer, 1977 Kuhns, 1979 Hakanson and Meredith, 1979 Scagliotti 1988 Hakanson et al., 1988 Brightman et al., 1989 Dorbandt et al., 2015 Gogova et al., 2020 Keenan et al., 2020 Cebrian et al., 2021 Mezzadri et al., 2021 Jacksz et al., 2021 Dulaurant et al., 2023	**805 C**, (40 F)	(fresh‐LCG) ABMG ±CPF ALCG CLCT CLCT‐MD CST CPF ± PBM TCPF CPF TCIG	**2 used 10–0, 9 used 9–0, 4 used 8–0, 2 used 7–0 and/or 1 used 6–0** (note: 4 studies used 2 sizes). **6 used SI, 5 used SI+SC or SI, 3 used SI or SC. 6 used PLG, 2 used PLGc 3 used PLG or PLGc 5 used NL 1 used SK AS > NAS**	**13 external 1 unreported 1 rotated** (note: in some of the cases in this article, the rest of the knots were external)	**12 cycloplegic/mydr.** (note: 7 atropine or cyclopentolate, 5 undetermined and simply reported as mydriatic or cycloplegic). **2 not mentioned**	**6 oral NSAID 1 oral NSAID + BL and TRPH 1 TRPH 1 “cut suture ends” 6 nothing more** (note: the 2 studies that used a TEF in one study and a TRPH in the other, reported the aim was to “support” and/ or “protect” the reconstruction)	**6 no mention. 2 as a definition in M&M**. **3 as an observation in the results** (note: with one of these in relation to the suture ends. causing discomfort). **3 in discussion** (note: 1 of these 3 also in the introduction)
***n* = 4**	**Donor tissue** Hacker, 1991 Hansen and Guandalini, 1999 Lacerda 2015 Voitekha and Shilkin, 2022	**135 C,** (97 F)	DLCG DFTCG FrFTCG FrLCG FzFTCG FzLCG	**3 used 9–0, 3 used 8–0 and/or 1 used 7–0** (note: 1 study used 3 sizes and 1 study used 2 sizes). **3 used SI 1 used SI+SC or SI 2 used PLG, 1 used PLG or PLGc, 1 used PLD, 4 used NL, and/or 1 used SK NAS > AS**	**4 external**	**4 cycloplegic/mydr.** (note: 2 atropine or cyclopentolate, and 2 tropicamide)	**1 oral NSAID 1 TEF only 1 TRPH only** (note: including partial and permanent TRPH) **1 nothing more**	**4 no mention**
***n* = 11**	**Biomaterials** Bussieres et al., 2004 Barros et al., 2005 Vanore et al., 2007 Goulle, 2012 Dulaurent et al., 2014 Chow and Westermeyer, 2016 Balland et al., 2016 Costa et al., 2019 Maini et al., 2020 Lavaud et al., 2021 Santillo et al., 2021	**332 C,** (107 F)	AM BP PBMs	**1 used 10–0, 10 used 9–0 and/or 3 used 8–0** (note: 3 studies used 2 sizes). 3 used SI, 5 used SI+SC or SI 1 used SI or SC (*), 2 pattern unknown (**) (*also used a FI and **1 of the 2 also used HOB). **7 used PLG 1 used PLGc 2 used NL 2 not reported but 1 stated it was AS AS > NAS**	**8 external 3 unreported**	**9 cycloplegic/mydr.** (note: 7 atropine, 1 atropine or cyclopentolate, and 1 cyclopentolate or tropicamide). **2 not mentioned**	**4 oral NSAID 2 oral NSAID + TEF 1 oral NSAID + TRPH 1 BL + TRPH 1 TEF 1 nothing more** (note: the 3 studies that used a TEF reported its use was to “support” the reconstruction with 1 of those 3 studies also aiming to reduce dehydration. The 1 study that used a BL and lateral TRPH reported the aim was to support the reconstruction and prevent dehydration. The 1 study that used a lateral TRPH reported it was used to protect the cornea)	**3 no mention of pain. 3 in introduction**, but mainly in relation to feline corneal sequestrum in cats*. **5 as an observation in the results** (note: and 2 of these 5 also in the introduction)
**Total. *n* = 29**		**1,272 C,** (244 F)		**3/38 (7.89%) counts used 10–0 22/38 (57.89%) 9–0, 10/38 (2.63%) 8–0, 2/38 (5.26%) 7–0, and/or 1/38 (2.63%) 6–0** (note: 9/29 (31.03%) studies used 2 or more sizes, with 8 of these using 2 sizes and 1 using 3 sizes, while 20/29 (68.97%) used 1 size). **12/28 (42.85%) used SI, 11/28 (39.29%) SI+SC or SI, 4/28 (14.29%) used SI or SC, 1/29 (3.45%) used FI, and 2/29 (6.9%) did not report it. 15/36 (41.67%) used PLG, 3/36 (8.33%) PLGc, 4/36 (11.11%)PLG/PLGc, 11/36 (30.56%) NL, 2 unreported but 1 stated it was AS, 2 SK, and 1 PLD** (note: 12/19 (63.16%) papers that used PLG did not report filament type, 4/19 (21.05%) reported using monofilament and 6–0 to 8–0 sizes 3/19 (15.8%) were counted as multifilament). **AS > NAS**	**25/29 (86.20%) external** (1/29 (3.45%) were rotated in some of the cases with the rest being external.) **4/29 (13.8%) unreported**	**25/29 (86.21%) had a cycloplegic/mydr.** (7 atropine, 10 atropine or cyclopentolate, 5 undetermined as simply a mydriatic or a cycloplegic, 2 tropicamide, and 1 tropicamide or cyclopentolate). **4/29 (13.79%) not mentioned**	**15/29 (51.72%) oral NSAID** (11 oral NSAID 4 oral NSAID + TEF or TRPH) **6/29 (20.68%) other** (2 TRPH only, 2 TEF only, 1 BL/TRPH only, and 1 “cut suture ends” only) **8/29 (27.59%) nothing more**	13/29 (44.83%) no mention. 3/29 (10.34%) in the introduction*. 2/29 (6.90%) in the materials and methods. 8/29 (27.59%) as an observation in the results, whether or not also mentioned in the introduction. 3/29 (10.34%) in the discussion.

*Note*: The number of studies is shown in the first column, where each reference is also shown in abbreviated form. This is followed by the number of eyes treated and the species canine (i.e., “C”) and feline (i.e., “F”) to show the results include feline cases in some studies, as they could not be separated. This is followed by the suture patterns and suture types used in each study, whether a study indicated if some or all the suture knots were external (i.e., as shown in photographs or a diagram for all or some of the sutures used, or because it was specifically mentioned), if a cycloplegic/mydriatic was used in the postoperative period, if there were other forms of pain control (i.e., the use of a bandage lens, tarsorrhaphy, third eyelid flap, an oral NSAID). The last column counts how many articles mentioned pain and/or discomfort or comfort, and where was it mentioned in the article (i.e., whether it was mentioned directly or indirectly (e.g., the use of a TEF for the control of pain) independently of it being mentioned in the introduction or discussion, or not). Bold letters show the most relevant data. The abbreviations are grouped by topic as follows: *Suture pattern* (FI, Ford interlock; SI, simple interrupted; SC, simple continuous; FI, Ford interlocking), *suture type* (PLG, polyglactin or Vicryl; PLGc, Polyglycolic acid; PLD, polydioxanone; SK, silk; NL, nylon; AS, absorbable suture; NAS, nonabsorbable usure), *autogenous material* (ABMG, autologous buccal mucous membrane grafts; ALCG, autogenous lamellar corneal graft; CIG, conjunctival island graft; CLCT, corneolimboconjunctival transposition (aka corneoconjunctival transposition, CCT); CLCT‐MD, when the CLCT is multidirectional; CST, Corneoscleral transposition; CPF, conjunctival pedicle flap; LCG, lamellar corneal graft; TCF, tarsoconjunctival flap; TCIG, tarsoconjunctival island graft; TEF, third eyelid flap (either used to protect a superficial keratectomy or specifically for control of postoperative pain depending on the column the entry is found)); *donor material* (DFTCG, dehydrated full‐thickness corneal graft, homologous or heterologous; DLCG, dehydrated lamellar corneal graft, homologous or heterologous; FrLCG, fresh lamellar corneal graft, homologous or heterologous, FrFTCG, fresh full‐thickness corneal graft, homologous or heterologous; FzFTCG, frozen full‐thickness corneal grafts, homologous or heterologous; FzLCG, Frozen lamellar corneal graft, homologous or heterologous); *biomaterial* (AM, amniotic membrane used either as a graft (i.e., sutured to the corneal wound) or a patch (i.e., sutured onto the limbus); BP, bovine pericardium; PBMs, porcine biomaterials (extracellular matrix of urothelium and small intestinal submucosa (i.e., BioSIS®); porcine urinary bladder submucosa (i.e., ACell Vet®), or acellular porcine corneal stroma (i.e., BioCorneaVet®))), *other* (BL. bandage lens; HOB, hydrogel ocular bandage (Ocuseal®); IM, intramuscular injection, Mydr., mydriatic; TRPH, tarsorrhaphy).

### 3.2 Use of corneal sutures in peer‐reviewed articles

Corneal suture size included five sizes spanning from 6–0 to 10–0 (Table 1). A total of 20/29 (68.97%) of the studies used only one suture size, while 9/29 (31.03%) studies used two or more suture sizes with 8/29 (27.58%) studies using two sizes and 1/29 (3.45%) using three different sizes. Considering that more than one suture size was used in several of the studies, there were 38 counts of suture size in the 29 articles included in the review, with 3/38 (7.89%) of these being for 10–0 suture, 22/38 (57.89) being for 9–0 suture, and 13/38 (34.21%) being for larger suture sizes (i.e., 6–0 to 8–0) with the majority of the latter (i.e., 10/38 (26.32%)) being for 8–0 suture. The use of 10–0 suture included three studies published in 1979, 1989, and 2016. The use of 9–0 suture included publications spanning from 1973 to 2023. While four counts of 9–0 suture included four studies published between 1973 and 1999, there were 18/22 (81.82%) counts for studies published between 2004 and 2023. Similarly, the use of 8–0 suture spanned from the late 70s to 2022, and while 4/10 (40%) of these counts included four studies published between the late 70s and the late 90s, the rest (i.e., 6/10 (60%)) included six studies published between 2004 and 2022. The two studies that used 7–0 suture were published in 1979 and 1988 and the single study that used 6–0 was published in 1977.

There was a total of 36 counts of suture material compared to the 38 counts of suture size because two of the studies that reported the suture size did not report the suture material, though one of the two revealed it was absorbable (Table 1). The commonest suture material used was polyglactin (15/36, 41.67%). There were 3/36 (8.33%) counts of polyglycolic acid use, and an additional 4/36 (11.11%) counts of polyglactin and/or polyglycolic acid use. The second commonest suture type was nylon, with 11/36 (30.56%) of the counts. There were two counts of silk and one of polydioxanone. When grouping the absorbable (i.e., polyglactin, polyglycolic acid, and polydioxanone) and nonabsorbable materials (i.e., nylon and silk), the commonest material used was absorbable (24/37, 64.86%). Absorbable suture material was also used more commonly in the “Patient's own tissues” and “Biomaterials” study groups (i.e., 20/29 (68.96%) counts vs. 8/29 (27.59%) counts of nonabsorbable material and 1/29 (3.45%) count where the nature of the material was not reported). The group with donor tissues had slightly more counts of nonabsorbable material 5/9 (55.56%) than absorbable material 4/9 (44.44%).

Although the most commonly used suture was polyglactin, a total of 12/19 (63.16%) of the studies that used this material did not report if it was monofilament or multifilament, while only 4/19 (21.05%) of the studies that reported it, mentioned it was monofilament (Table 1). The suture size reported in the remaining 3/19 (15.8%) articles was 6–0 to 8–0, and these are typically multifilament sutures.

A total of 27/29 (93.10%) of the articles reported the suture pattern used. A simple interrupted pattern was used in 23/27 (85.19%) of the articles in all or some of the cases, with 12/27 (44.44%) using exclusively a simple interrupted pattern, and 11/27 (40.74%) using a mixture of simple interrupted and simple continuous patterns or only simple interrupted sutures. While simple continuous sutures (i.e., with or without simple interrupted sutures) were used in some or all of the cases in 15/27 (55.55%) of the studies, only 4/27 (14.82%) of the studies reported using exclusively simple continuous sutures in an undetermined number of cases.

None of the studies indicated the knot configuration used, none of the articles included the number of suture knots used in each reconstruction, and 28/29 (96.55%) articles did not report if the suture knots were external or if an attempt was made to bury the knots. One article mentioned that the patients of one of the authors had all the simple interrupted suture knots of 9–0 polyglactin suture turned into the suture track in an attempt to reduce postoperative eyelid and third eyelid irritation during blinking and/or ocular movement. However, the specific number of patients with buried knots was not reported. A total of 25/29 (86.20%) of the articles showed images that demonstrated external knots had been used in at least some of the cases. The rest (4/29 (13.79%)) did not show images of the surgeries and did not report if external or buried knots were used.

### 3.3 Use of microsutures in textbooks and single‐topic journals

One single‐topic journal that included two chapters^46^, ^47^ and two textbooks written in English that included several chapters dedicated to general concepts of ophthalmic microsurgery and /or corneal microsurgery for small animals were found.^44^, ^45^, ^48^ The single‐topic journal focused on ocular surgery and contained two chapters with passages in which suture knot burial was mentioned. The first chapter reported that “very small suture” knots may be buried to avoid palpebral irritation during blinking.^46^ The second chapter recommended the use of simple interrupted microsutures for corneal reconstruction and mentioned the possibility of burying corneal knots of sutures smaller than 9–0 and a preference for 10–0 suture for keratoplasty because the “suture knots could be buried.”^47^ While the topic of knot burial was mentioned, the techniques to bury the suture knots were not included. One of the ophthalmology textbooks was not exclusively a surgical book but met the inclusion criteria because it contained a chapter on corneal diseases and corneal surgery.^44^ The diagrams depicted the use of simple interrupted sutures with external knots.^44^ The chapter on microsurgical principles of a veterinary ophthalmology surgical text mentioned the possibility of burying corneal knots of 9–0 or smaller suture by “*snugging the knot into the suture*” track.^49^

### 3.4 The reporting of discomfort/pain and the medication used to control it in peer‐reviewed articles

A total of 13/29 (44.83%) of the articles did not mention pre‐ or postoperative discomfort/pain (Table 1). The introduction of 3/29 (10.34%) of the articles contained a mention of discomfort/pain being associated with the disease process being treated, but it only related to the cats with feline corneal sequestrum included in the studies. Two out of 29 (6.90%) of the articles mentioned discomfort/pain in the materials and methods (e.g., “reaching comfort” was the point at which the use of atropine could be stopped). Eight out of the 29 articles (27.59%) mentioned discomfort/pain as an observation in the results, whether or not it was also mentioned in the introduction, while 3/29 (10.34%) of the articles mentioned it in the discussion.

A total of 25/29 (86.21%) of the articles reported the use of a cycloplegic/mydriatic as part of the postoperative care and the rest (i.e., 4/29 (13.8%)) did not (Table 1). While 17/25 (68%) of the articles reported the cycloplegic/mydriatic chosen was atropine or cyclopentolate, in 5/25 (20%) of the articles the type of drug was not specified, and in 3/25 (12%) tropicamide was chosen for all the cases (i.e., two studies) or some of the cases (i.e., one study, with an unknown percentage of the cases in this study given cyclopentolate) (Table 1). Lastly, while 15/29 (51.72%) of the studies reported administering an oral NSAID for the early postoperative period (i.e., between 3 to up to 14 days) whether or not this also included the use of a lateral tarsorrhaphy or third eyelid flap, 8/29 (27.59%) of the articles included no pain medication as part of the aftercare (Table 1). Six out of 29 of the studies (20.68%) reported the use of a tarsorrhaphy, a bandage lens and a tarsorrhaphy, or a third eyelid flap. However, all of those studies reported doing this to help protect the corneal reconstruction and/or to help prevent corneal dehydration and did not clarify if the use of these measures was also intended to be the control of ocular discomfort/pain (Table 1).

## 4 DISCUSSION

It is important to have an overview of the technical aspects common to most corneal surgeries to gain a deeper understanding of the factors that might affect the choice of corneal reconstruction, corneal healing, and patient comfort. It is also important to understand if the recommendations made in surgical texts are reflected in the clinical choices made. The present work reviews the veterinary literature on the topic of canine corneal reconstruction spanning the last 60 years, with the goal to summarize the use of corneal suturing techniques for corneal reconstruction in dogs, to identify areas where key information might be missing from studies, and to highlight the presence of knowledge gaps that deserve further attention. The latest edition of veterinary ophthalmology textbooks with part of the book dedicated to general microsurgical techniques for ophthalmic use and corneal reconstruction, and single‐topic journals with an issue on the same topics were included.

The review revealed that absorbable sutures were used more commonly than nonabsorbable sutures. Similarly, simple interrupted sutures were used more commonly, whether on their own or when more than one type of suture pattern was used. Interestingly, none of the reports included the type of knot configuration used or the median/range number of knots used per case. Yet, it would be reasonable to assume that because a simple interrupted pattern was commonly used, numerous corneal repairs must have had several suture knots, and that knot tying was an important technical part of the repair. Therefore, it would seem reasonable to encourage record keeping and reporting of all of these relatively straight forward suture parameters.

The review of the surgery texts revealed that details of specific techniques to bury corneal knots were unreported though the burial of corneal knots were occasionally mentioned.^45^, ^46^, ^47^, ^49^ Three of the texts briefly mentioned the possibility of burying the knots into the suture track,^45^, ^46^, ^49^ while only one referred to a knot burial technique by name (i.e., the “snugging” of suture knots into the suture track).^49^ The latter refers to the turning of simple interrupted suture knots into the suture track and is the same as to what is referred to in the present review as the “tugging technique.”^49^ Similarly, only one article in the review mentioned the “tugging” of knots of 9–0 monofilament polyglactin material to improve patient comfort postoperatively.^18^ While it is possible that some of the surgeons in the other articles buried some or even all of the corneal knots, this was not reported, which made it impossible to draw further conclusions. Veterinary patients feel corneal knots found on the ocular surface during blinking and ocular movement as they rub on the conjunctival surfaces, and this is known to be a potential source of patient discomfort.^45^, ^46^, ^49^ Therefore, it would be sensible to expect corneal surgeons to opt for a small suture. Despite this, numerous articles reported using 8–0 and the use 10–0 was rarely reported. As suggested in at least one of the textbooks, it would seem sensible to encourage the use of the smallest suture possible if the surgeon decided to leave corneal knots on the ocular surface, as this will lead to a smaller knot size, and that in the case of polyglactin 9–0 that the surgeon ensures the thread is monofilament.^45^ In addition, it would also seem sensible to encourage all surgeons using 9–0 and 10–0 sutures to make use of knot burial techniques. While some microtrauma might result from the “tugging” technique, and this might be more relevant when burying simple interrupted knots of 9–0 monofilament material compared to 10–0 material, there are no studies that describe if the “tugging” technique with these suture sizes might affect corneal healing in dogs, an area that deserves further study. There are other techniques for the burial of corneal sutures that would, in principle, avoid this type of microtrauma. One such technique is the “deep‐superficial‐superficial‐deep technique,” which may be used for simple interrupted sutures, while its modification may be used to bury the knots at the start and the end of a simple continuous suture pattern (unpublished data from the author, and personal communication Dr. Larry Benjamin FRCS(Ed), FRCOphth, DO and Dr. Jeniffer Watts MB Bchir MA FRCS FRCOpth) (Table 2). None of the articles included in the review reported using these other methods, making it impossible to know if the mention of these burial techniques was simply omitted, if the techniques were unfamiliar to the surgeons, or if the surgeons purposely avoided them and, if so, why. However, an infrequent mention in textbooks and other sources of literature suggests knot burial techniques might not be widely used by or be widely known to veterinary corneal surgeons.

**TABLE 2:** There are three relatively simple ways in which a corneal surgeon can ensure all the corneal knots are buried, and they are listed and briefly described here.

Technique	“Tugging” technique	Deep‐superficial‐superficial‐deep (“DSSD”) technique/suture pattern	Adaptation of the “DSSD”
Suture pattern	Simple interrupted (SI)	Simple interrupted (SI)	Simple continuous (SC)
Knot configuration	2–1–1	2–1–1	2–1–1 (at the start and at the end)
Description	The surgeon tugs on the suture so as to rotate it and force the knot into the suture track. Arguably, might work best when the knot is turned into the point of entry of the needle (i.e., rather than the point of exit). Difficult with small sutures (i.e., <1 mm on either side of the wound)	The surgeon starts and ends the suture bite on the inside of the wound, so that when the knot is tied it remains buried	Starts as the “DSSD” technique for SI sutures but uses the long end of the suture to create a SC suture that is tied at the end with a buried knot

The review revealed inconsistencies in the reporting of ocular discomfort/pain. While approximately half of the articles mentioned it, very few mentioned it as an observed result, and a small proportion of the articles discussed it. Corneal ulcerative disease affects brachycephalics and spaniel type dogs disproportionately compared to mesaticephalic and dolichocephalic breeds.^50^ While brachycephalic dogs have been reported to have lower corneal sensitivity as measured by corneal aesthesiometry,^51^ there is no evidence to back a potential assumption that a lack of reporting of discomfort/pain necessarily means there was an overrepresentation of brachycephalic breeds, though this remains a possibility. Conversely, there is no evidence to support a potential assumption that there was discomfort/pain, but surgeons failed to report it. The reasons why discomfort/pain was reported so rarely remains unknown. Interestingly, most of the articles included in the review reported the use of a cycloplegic/mydriatic and half of the studies reported the use of an oral NSAID as part of the postoperative care of the patients. These findings imply that, in at least some of the patients, the surgeons took steps to minimize pain associated with reflex uveitis, keratitis, and/or the feeling of superficial corneal knots during blinking and ocular movement.

An ocular pain scoring that veterinary ophthalmologists may rely on does not exist, and this might have also affected the reporting of ocular discomfort/pain in the studies included in this review. However, there are clinically visible signs commonly associated with eyelid, conjunctival, corneal and uveal pain (i.e., blepharospasm and tearing) that surgeons could report on.^52^, ^53^, ^54^, ^55^ Even if an observation of discomfort/pain is subjective, it could be argued that avoiding making such an observation could lead to the impression that there is no discomfort/pain, or that patient comfort is not an important part of CUD and corneal reconstruction. Therefore, it would be prudent for surgeons to include a subjective clinical observation of signs commonly associated with patient discomfort/pain than to include no observation at all. Subjective observations must be treated with great caution, as they can lead to erroneous conclusions, and should always be clearly identified as such (i.e., by clearly labeling it a “subjective clinical observation of ocular discomfort”). As an example, surgeons could report on the presence/absence of an increased blink, blepharospasm, and/or tearing compared to the unaffected/healthy eye (or compared to a non‐painful eye if both eyes were affected or in patients with only one eye), and they could report on the effect of a topical anesthetic drop on these signs (i.e., after the onset of action of the drop), in ophthalmic examinations while still acknowledging there is no “golden standard” to reliably assess ocular pain in dogs. These observations should be made before and after surgery.

### 4.1 Limitations

Some of the limitations that affect the data in the present review are inherent to the nature of literature review, which included all the limitations of each of the studies (e.g., missing data, cases lost to follow up, low number of cases in some groups, and mixing of results of dogs and cats in some studies, among other things). It is also possible that the literature review might have unintentionally omitted some articles. However, the ones included make the bulk of the available literature on this topic and, as such, serve as a general overview.

## 5 CONCLUSIONS

A review of the peer‐reviewed veterinary literature written in English on the topic of corneal reconstruction for CUD in dogs revealed that there were inconsistencies in the reporting of corneal sutures and suturing techniques used, and the reporting of possible ocular pain. Recommendations may be made for surgeons to record and report the suture type, suture pattern, knot configuration and if buried knots were used in addition to the technique used to bury them. While there is no reliable corneal pain scoring system for use in dogs, surgical reports could include a subjective, clinical appreciation of the presence of signs often seen in painful ocular conditions before and after surgery, and if these improve with the use of a topical anesthetic. Further studies are needed on the use of buried knots for corneal reconstruction in dogs, and on the subject of ocular pain in CUD before and after reconstructive surgery.

## AUTHOR CONTRIBUTIONS

The development of the idea, as well as the writing and editing, was carried out by the author.

## CONFLICT OF INTEREST STATEMENT

The authors confirm there are no conflicts of interest.

## ETHICS STATEMENT

This study complies with the Guidelines for Ethical Research in Veterinary Ophthalmology (GERVO) and is exempt from approval by an ethics committee. All the owners of client owned animals whose clinical data are included gave permission for the use of the clinical data for studies.

## Data Availability

Data sharing not applicable to this article as no datasets were generated or analyzed during the current study. All the reviewed articles are part of the veterinary and medical literature.
